# Integrated home-based screening for people living with disabilities: A case study from rural Malawi

**DOI:** 10.4102/ajod.v8i0.582

**Published:** 2019-11-22

**Authors:** Elizabeth M. Petersen, Emily B. Wroe, Kondwani Nyangulu, Chisomo Kanyenda, Sam Njolomole, Elizabeth L. Dunbar, Chiyembekezo Z. Kachimanga

**Affiliations:** 1 Division of Global Health Equity, Brigham and Women’s Hospital, Boston, United States; 2 Partners In Health, Neno, Malawi; 3 Partners In Health, Harper, Liberia

**Keywords:** Malawi, disability, screening, task shifting, primary healthcare

## Abstract

People living with disabilities (PLWDs) have poor access to health services compared to people without disabilities. As a result, PLWDs do not benefit from some of the services provided at health facilities; therefore, new methods need to be developed to deliver these services where PLWDs reside. This case study reports a household-based screening programme targeting PLWDs in a rural district in Malawi. Between March and November 2016, a household-based and integrated screening programme was conducted by community health workers, HIV testing counsellors and a clinic clerk. The programme provided integrated home-based screening for HIV, tuberculosis, hypertension and malnutrition for PLWDs. The programme was designed and implemented for a population of 37 000 people. A total of 449 PLWDs, with a median age of 26 years and about half of them women, were screened. Among the 404 PLWDs eligible for HIV testing, 399 (99%) agreed for HIV testing. Sixty-nine per cent of PLWDs tested for HIV had never previously been tested for HIV. Additionally, 14 patients self-reported to be HIV-positive and all but one were verified to be active in HIV care. A total of 192 of all eligible PLWDs above 18 years old were screened for hypertension, with 9% (*n* = 17) referred for further follow-up at the nearest facility. In addition, 274 and 371 PLWDs were screened for malnutrition and tuberculosis, respectively, with 6% (*n* = 18) of PLWDs referred for malnutrition, and 2% (*n* = 10) of PLWDs referred for tuberculosis testing. We successfully implemented an integrated home-based screening programme in rural Malawi.

## Introduction

People living with disabilities (PLWDs) constitute up to 15% of the global population, and about 80% of them live in low- and middle-income countries (Hanass-Hancock et al. 2016). It is well documented that PLWDs have poor access to healthcare, a problem that is worse in rural than urban areas (World Health Organization [WHO] 2011). For sexual and reproductive health services (SRHS), PLWDs are often not involved in planning nor targeted for routine treatment and screening. Healthcare workers are often not trained on how to provide services to PLWDs, and some deny them care as they consider PLWDs asexual (Hanass-Hancock 2009). Although the disability itself may prevent access to care, some studies also attribute access difficulties to poor socio-economic status (Eide et al. 2015; Vergunst et al. 2017). For example, in many African countries, including Malawi, the main reasons for lack of access to health services for PLWDs include lack of transport, inadequate availability of services, drugs and equipment, and high out-of-pocket costs (Eide et al. 2015). These challenges are magnified by a weak health system, poverty, illiteracy, stigma and marginalisation of PLWDs (Hanass-Hancock, Regondi & Naidoo, 2013; Vergunst et al. 2017).

Caregivers and families of PLWDs also face challenges with accessing health services. Households with PLWDs are worse off: they tend to have a high number of dependents, high unemployment, a lower income, reduced access to education and in general have higher levels of poverty (Munthali 2011).

People living with disabilities are a vulnerable population, and healthcare systems should be adapted to address their barriers to care. With the shortage of professional healthcare workers in many low-income countries, including Malawi (Malawi Government, 2017), new approaches for delivering services in the community and household need to be developed. One approach is task shifting, a process of transitioning complex responsibilities to less specialised staff. While task shifting has been successful in many SRHS, including HIV programmes (Callaghan, Ford & Schneider 2010; Dawson et al. 2014), programmes need to adapt task shifting for home-based screening, focusing on appropriate supervision, well-trained staff and appropriate allocation of responsibilities to ensure quality services (Dawson et al. 2014).

Neno District is a southwestern district of Malawi with an estimated population of 150 800 (National Statistical Office 2008). Recent estimates showed that approximately 5000 people, or 3.5% of the population, are living with disabilities (Partners In Health 2015). This case study reports an approach to home-based screening for HIV, malnutrition, tuberculosis and hypertension for PLWDs and their families in Neno District.

## Setting and approach

The PLWDs home-based screening programme was instituted in response to a 2014 survey conducted by community health workers (CHWs) to identify the number of PLWDs in Neno District. The CHWs, who are all long-term residents of Neno and have been employed since 2007, focus on education, case identification and referral for HIV and tuberculosis services. During the survey, PLWDs expressed significant challenges in accessing screening services because of lack of transport and lack of health service prioritisation for PLWDs at the facilities.

To address these challenges, in 2016, this programme was piloted in a remote area on the Mozambique border, targeting an estimated 1300 PLWDs of the population of 37 000 people (Partners In Health 2015). Neno is one of the poorest and most remote districts of Malawi, with a poverty level 15% higher than the national average (National Statistical Office 2012). The goal was to revisit households that registered PLWDs during the survey and offer screening services. Additionally, efforts were made to identify PLWDs not originally identified. As in the initial survey, PLWDs were identified based on the International Classification of Functioning, Disability and Health (ICF), which encompasses impairments, activity limitations and participation restrictions (WHO, 2018). People living with disabilities were identified by asking disability-related questions that identified functional limitations in the three domains of ICF (Box 1). Based on the screening questions, people were classified as either having a disability or not; no grading of disability was performed.

BOX 1 Screening questions for disabilities.

1. Do you or a member of the household have difficulty seeing, even if wearing glasses?
2. Do you or a member of the household have difficulty hearing, even if using hearing aid?
3. Do you or a member of the household have difficulty walking or climbing steps?
4. Do you or a member of the household have difficulty remembering or concentrating?
5. Do you or member of the household have difficulty with self-care, such as washing all over or dressing?
6. Using your or their usual (customary) language, do you or a member of the household have difficulty communicating, understanding or being understood?

Each question had the following responses: (1) No, no difficulty. (2) Yes, some difficulty. (3) Yes, a lot of difficulty. (4) Cannot do it at all.

*Source*: United Nations Washington Group on Disability Statistics, 2016, ‘Short set of disability questions’, United Nations Washington Group on Disability Statistics, viewed 20 September 2018, from http://www.washingtongroup-disability.com

To maximise integration, several screening programmes were combined, namely HIV, tuberculosis, malnutrition and hypertension (Table 1). Because of shortage of clinical staff, task shifting for home-based screening utilised three groups of support staff: CHWs on a monthly stipend, one clerk from the HIV clinic and HIV testing counsellors. Community health workers personally knew the PLWDs and were responsible for informing community leaders and households at least a day before the screening. Additionally, as part of their routine tasks, CHWs were already trained to provide malnutrition and tuberculosis screening. For HIV screening, existing HIV counsellors provided counselling, testing and referral. These counsellors were already working in the district and received a standard 4-week training organised by Malawi Ministry of Health. The clerk who normally worked in an HIV clinic providing routine screening provided hypertension, tuberculosis and malnutrition screening and supervised the team. None of the staff were providing all four screenings as HIV screening required nationally certified HIV providers. This multidisciplinary team received a 2-day additional training on identification of PLWDs, appropriate communication to PLWDs and screening and referral procedures for different conditions. All screening was provided free of charge.

**TABLE 1:** Screening and referral criteria for home-based screening.

Disease condition	Health services	Testing and referral eligibility criteria	Equipment used	Referral pathway
HIV	Individual HIV counselling (pre-test and post-test counselling) according to national guidelines at the time. Whole blood rapid antibody HIV test	Over 2 years,^‡^ undocumented HIV test result or HIV-negative test over 3 months, do an HIV test If known HIV-positive, refer to health facility if they are not enrolled or had missed their last appointment	Supplied by Ministry of Health Based on the Ministry of Health guidelines at the time, equipment include HIV rapid test kits (Determine and UniGold tests), buffer, lancets and pipettes	To nearest primary facility for enrolment in care
Tuberculosis	Asked the cardinal signs of tuberculosis Cough over 2 weeks if HIV-negative Any cough if HIV-positive Weight loss	All age groups Collect sputum sample the same day of screening. If unable to collect sputum sample, refer the client to nearest facility	Sputum bottles National tuberculosis forms Sputum collection box	Nearest primary facility
Malnutrition	Measurement of mid-upper arm circumference (MUAC) from 6 months to 18 years old	Based on national guidelines, refer as follows 6 months to 4 years-MUAC less than 12.5 or oedema 5–9 years- MUAC less than 14.6 or oedema 10–14 years-MUAC less than 18.6 or oedema 15–18 years- MUAC less than 22.0 18 years and above-BMI based on national protocol BMI less than 18.5	MUAC tapes Height board Weighing scale Batteries BMI wheel	Primary care clinics
Hypertension	Measurements of blood pressure using an automated blood pressure machine	19 years and above Refer if BP over 160 and/or 110 on a single reading†	Automatic blood pressure machine Batteries	Nearest primary facility for confirmation and enrolments with NCD care clinics†

*Source*: Partners In Health, 2015, *Neno district people with disabilities needs assessment: Key findings*, Partners In Health, Neno

Note: Other materials needed across all screening – referral slips, alcohol swabs, hand sanitizers, gloves and writing materials.

HIV, human immunodeficiency virus; BMI, body mass index; NCD, non-communicable diseases.

† , This threshold was clinical protocol at that time in Neno, as chronic care services were being rolled out in a stepwise fashion to avoid overwhelming the fledgling clinics with mild disease.

‡ , Because of the presence of maternal antibodies in children less than 2 years, HIV antibody tests were not recommended for children less than 2 years during the time of the programme. The recommended test was HIV DNA test that was performed in a central laboratory over 2 h away from the district. As we were using HIV antibody rapid test in this programme, we excluded children who were less than 2 years from HIV screening.

Household-based screening started in March 2016. Consent to screen households for PLWDs was obtained from the head of household. Additional informed consent was obtained from PLWDs if they were over 18 years and fit to give consent, and if not, informed consent was obtained from a legal guardian. To ensure confidentiality, all screening and communication of results was conducted in a private space within the household. If PLWDs or household members screened positive or were seen to be sick, they were counselled on-site about their condition by a team member and referred to the nearest facility. Since households with PLWDs often have barriers in accessing healthcare services, the screening was offered to all household members who were given a choice to opt out of any of the screening services, although the intervention prioritised screening PLWDs.

As the screening team was already working with patients, they were reminded on infection prevention and given materials for infection prevention. This home visit package is explained in Table 1. All results were recorded in a specially designed screening register and were transferred to Excel for cleaning and analysis.

## Relevant changes

Between March and November 2016, 600 people were screened in their homes. Among the individuals screened, 75% (*n* = 449) were PLWDs and the remaining were their family members. The median age of PLWDs was 26 years (IQR 13–51), 48% (*n* = 217) were women and 70% (*n* = 315) were 15 years and above (Tables 2 and 3).

**TABLE 2:** Types of disabilities.

Disability type	Number	Percent
Physical illnesses except epilepsy	227	50.6
Epilepsy	52	11.6
Vision impairment	38	8.5
Hearing impairment	32	7.1
Communication unrelated to hearing impairment	19	4.2
Anosmia (inability to perceive smell)	2	0.5
Intellectual or mental health disabilities	15	3.3
Multiple disabilities†	11	2.5
Activity limitation	22	4.9
Participation restriction	5	1.1
Un-specified	26	5.8

**Total**	**449**	**100.0**

† , Patients in this group had multiple functional disabilities: impairment and/or activity limitation and/or participation restriction.

**TABLE 3:** Characteristics of patients screened using home-based integrated testing.

Variables	Total		PLWDS		Other family members	
	*n*	%	*n*	%	*n*	%
Individuals screened	600	-	449	7	151	25
Median age	30	16,52†	26	13, 51†	39	25, 55†
**Gender**						
Males	261	-	232	52	29	19
Females	339	-	217	48	122	81
**HIV screening**						
Total screened for HIV	-	-	-	-	-	-
Already HIV-positive	15	-	14	-	1	-
Known HIV-negative	25	-	12	-	13	-
Offered an HIV test	531	-	404	-	127	-
Successfully tested for HIV at home	525	-	399	-	126	-
Tested for HIV: New HIV-positive	3	-	1	-	2	-
Tested for HIV: new HIV-negative	522	-	398	-	124	-
**Hypertension screening**						
Screened for hypertension	296	-	192	-	104	-
Referred for hypertension	28	9	17	9	11	11
**Nutrition screening (Eligible)**						
Screened for nutrition	367	-	274	-	69	-
Under nutrition	24	6	18	6	6	8
**Tuberculosis screening**						
Total screened	496	-	371	-	125	-
Total referred	12	2	10	2	2	2

HIV, human immunodeficiency virus; PLWDs, people living with disabilities.

† , Interquartile range.

Of the 449 PLWDs screened for HIV, only 6% (*n* = 26) of those eligible for testing had a known HIV status (14 were HIV-positive and 12 had a negative test result within the past 3 months). Among those who were HIV-positive (*n* = 14), all except one individual were enrolled in HIV care.

Among all remaining PLWDs (*n* = 404), three people were ineligible for an HIV test as they were aged less than 2 years, and two individuals declined testing. Of those eligible for an HIV test, 99% (*n* = 399) accepted the HIV test. Among the 399 tested successfully, 69% (276) never had an HIV test before. At the end of the screening, one person tested HIV-positive and was successfully linked to care.

In total, 192 of the 280 eligible PLWDs above 18 years old were screened for hypertension, with 9% (*n* = 17) referred for follow-up for blood pressures greater than 160 mmHg systolic or 110 mmHg diastolic. An additional 274 and 371 PLWDs were screened for malnutrition and tuberculosis, respectively. Six per cent (*n* = 18) of those screened for malnutrition were referred to care, as were 2% (*n* = 10) of those screened for tuberculosis.

## Lessons learnt

As Malawi is struggling with a double burden of HIV and non-communicable diseases, programmatic approaches are needed to reach key populations where they live (Malawi Government 2017). Because of a shortage of health workers, it may not be possible to use formally trained professionals to provide household-based screening. Limited information describing the use of task shifting to identify PLWDs and provide household screening exists in Malawi. Household-based studies report on disability identification and linkage to care by CHWs and/or non-clinical staff (Mulwafu et al. 2017; Tataryn et al. 2017). We could not find any programme that employed task shifting to provide integrated household screening, including hypertension screening, for PLWDs.

Secondly, the programme demonstrated the feasibility and acceptability of combining screening for HIV and other conditions, including hypertension, in a household setting: a high proportion of PLWDs accepted the services. However, few family members accepted to be screened. The screening team focused screening the PLWDs and it may be the reason that influenced lower uptake, but we were not able to collect information on why family members and some PLWDs refused some of the screening. This could be addressed in programmes similar to this case study.

Thirdly, this project provided one approach for household-based targeted disease screening, specifically for HIV, to a vulnerable population. People living with disabilities were targeted because of their limited access to services. Up to 10% of the PLWDs were referred for services. A large majority of the PLWDs had never before had an HIV test, which is striking given the considerable investments on HIV programme in Malawi. We hypothesise that this could be the result of stigma and difficulty accessing available services related to disability (Mcbain et al. 2017). Further efforts will be required to ensure that patients who tested negative remain negative; this will be addressed through continued preventive services by the CHWs. After the pilot, we could not roll out the programme to the whole district because of limited funding; however, with funding, this could be scaled up to other settings, and considering the specific needs of PLWDs is a component of ongoing exploration in Neno for the optimisation of integrated home visits.

There are a few notable limitations to this programme. Firstly, the disability identification tool we used mainly identified self-reported functional disability. Additionally, this identifies the most common disabilities and may miss ‘invisible disabilities’, such as mental health disorders. We also could not measure the severity of the disabilities. Secondly, the programme was carried out with a small population in a remote area of Malawi, making it difficult to generalise to Neno and other district of Malawi. Thirdly, we were not able to cost this programme that would give more information in whether scale-up would be recommended. Fourthly, we referred patients to the nearest health facilities but were not able to ascertain if all PLWDs were successfully linked to care.

Future programmes would consider the following steps to improve on our limitations: (1) expand household screening to other common chronic condition such as diabetes as they are also common in low- and middle-income countries, (2) cover a larger population to identify more PLWDs who can benefit from the screening, (3) design efforts that can be made to ensure that many family members of PLWDs are screened and document reasons of refusals for both PLWDs and family members, (4) measure costs to help other programme managers to plan and implement this programme and (5) design strategies to ensure that referred PLWDs receive the care at the facility as they may either not go to facilities or face other barriers once they arrive at the facility.

NB: The disability-associated questions were designed by United Nations Washington Group on Disability Statistics (http://www.washingtongroup-disability.com). They were translated to local language by bilingual speaker (both English and Chichewa speaker [local language of Malawi]) and were pre-tested in the initial survey conducted in 2014. The people screened were classified as either having disability (if they have a score 2 or above) or no disability if they score 1. The type of disability was also recorded.
